# Absence of knockdown resistance suggests metabolic resistance in the main malaria vectors of the Mekong region

**DOI:** 10.1186/1475-2875-8-84

**Published:** 2009-04-28

**Authors:** Katrijn Verhaeghen, Wim Van Bortel, Ho Dinh Trung, Tho Sochantha, Marc Coosemans

**Affiliations:** 1 Department of Parasitology, Institute of Tropical Medicine, Nationalestraat 155, B-2000 Antwerp, Belgium; 2 Department of Entomology, National Institute of Malariology, Parasitology and Entomology, Luong The Vinh Street, B.C. 10.200 Tu Liem, Hanoi, Vietnam; 3 Department of Entomology, National Center for Malaria Control, Parasitology and Entomology, 372 Monivong Boulevard, Phnom Penh, Cambodia; 4 Department of Biomedical Sciences, Faculty of Pharmaceutical, Veterinary and Biomedical Sciences, University of Antwerp, Universiteitsplein 1, B-2610 Antwerp, Belgium

## Abstract

**Background:**

As insecticide resistance may jeopardize the successful malaria control programmes in the Mekong region, a large investigation was previously conducted in the Mekong countries to assess the susceptibility of the main malaria vectors against DDT and pyrethroid insecticides. It showed that the main vector, *Anopheles epiroticus*, was highly pyrethroid-resistant in the Mekong delta, whereas *Anopheles minimus sensu lato *was pyrethroid-resistant in northern Vietnam. *Anopheles dirus sensu stricto *showed possible resistance to type II pyrethroids in central Vietnam. *Anopheles subpictus *was DDT- and pyrethroid-resistant in the Mekong Delta. The present study intends to explore the resistance mechanisms involved.

**Methods:**

By use of molecular assays and biochemical assays the presence of the two major insecticide resistance mechanisms, knockdown and metabolic resistance, were assessed in the main malaria vectors of the Mekong region.

**Results:**

Two FRET/MCA assays and one PCR-RFLP were developed to screen a large number of *Anopheles *populations from the Mekong region for the presence of knockdown resistance (*kdr*), but no *kdr *mutation was observed in any of the study species. Biochemical assays suggest an esterase mediated pyrethroid detoxification in *An. epiroticus *and *An. subpictus *of the Mekong delta. The DDT resistance in *An. subpictus *might be conferred to a high GST activity. The pyrethroid resistance in *An. minimus s.l*. is possibly associated with increased detoxification by esterases and P450 monooxygenases.

**Conclusion:**

As different metabolic enzyme systems might be responsible for the pyrethroid and DDT resistance in the main vectors, each species may have a different response to alternative insecticides, which might complicate the malaria vector control in the Mekong region.

## Background

In the Mekong region, the malaria vector control relies on the use of insecticides for impregnation of bed nets and for indoor residual spraying and is mainly focussed on three vector species complexes: *Anopheles dirus sensu stricto *(*s.s*.) [1], *Anopheles minimus sensu lato *(*s.l*.) [2] and *Anopheles sundaicus s.l*. [3]. Hence, the emergence of insecticide resistance in vector species may have important implications for the effectiveness of insecticide based vector control measures. This is particularly true for the Mekong region, where vector control efforts have significantly contributed to decrease the malaria burden in recent years [4].

WHO bioassays done in the framework of a three year survey (from March 2003 until July 2005) on insecticide resistance in the Mekong region (Vietnam, Laos, Cambodia and Thailand) have shown that different levels of pyrethroid and DDT resistance occur in the main malaria vectors of the Mekong region [5]. *Anopheles dirus s.s*., the main vector in forested malaria foci, was permethrin susceptible throughout the region. In central Vietnam, it showed possible resistance to type II pyrethroids. In the Mekong Delta, *Anopheles epiroticus *was highly pyrethroid-resistant. It was susceptible to DDT, except near Ho Chi Minh City where it showed possible DDT resistance. In Vietnam, pyrethroid-susceptible and tolerant *An. minimus s.l*. populations were found, whereas the *An. minimus s.l*. populations of Cambodia, Laos and Thailand were susceptible [5].

As each insecticide resistance mechanisms may have a different impact on the effectiveness of the pyrethroid-based control programmes, knowledge on the involved pyrethroid resistance mechanisms is necessary to guide the insecticide use in the vector control programmes. A resistance mechanism against pyrethroids and DDT, known as knockdown resistance (*kdr*), has been linked to mutations in the *para*-type sodium channel gene. Knockdown resistance has been described in several insect species [6]. In the African malaria vector, *Anopheles gambiae s.s*., two substitutions at codon 1014 (L1014F and L1014S) of domain II of the sodium channel gene have been associated with knockdown resistance [7,8]. Beside knockdown resistance, metabolic resistance mechanisms have been found in pyrethroid- and/or DDT-resistant *Anopheles *populations. Biochemical assays have been developed to measure levels of monooxygenases, esterases and glutathione-S-transferases (GST) in mosquitoes and elevated levels of such enzymes may enhance insecticide tolerance in *Anopheles *populations [9-11]. The present study aims to explore the involvement of knockdown and metabolic resistance mechanisms in providing DDT and pyrethroid resistance in the main malaria vectors of the Mekong region.

## Methods

### Mosquito samples

**Figure 1:**
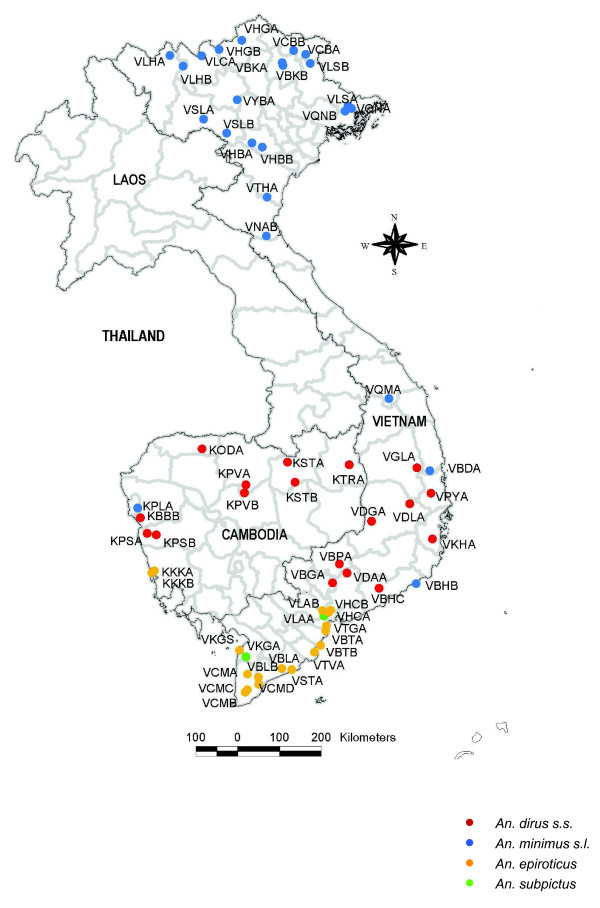
**Map showing the *An. minimus s.l*., *An. epiroticus*, *An. dirus s.s*. and *An. subpictus *populations analysed in this study**. All *An. minimus s.l*., *An. epiroticus *and *An. subpictus *populations, except the VHBA, VHBB and VSLB *An. minimus *populations, were resistant or possible resistant to DDT and/or pyrethroids. Although, the *An. dirus s.s*. populations were DDT and pyrethroid susceptible, these populations showed high levels of variation in mean knockdown time (KDT50) (ranged from 8 till 31 minutes for DDT and from 8 till 24 minutes for pyrethroids). Mosquitoes of the *Anopheles *populations indicated on the map were further analysed for the presence of *kdr *mutations. The pyrethroid and DDT resistant *An. minimus s.l*., *An. epiroticus *and *An. subpictus *populations of Vietnam were additionally subjected to biochemical assays.

In the framework of a three-year survey (from March 2003 until July 2005) on insecticide resistance in the Mekong region (Vietnam, Laos, Cambodia and Thailand), adult mosquitoes were collected by different collection methods (indoor and outdoor human landing collection, collection on cattle and morning resting collections inside houses) and their susceptibility was assessed against DDT and pyrethroids by use of the WHO bioassays. The bioassay results were summarized in three resistance classes as defined by WHO [12]: (1) susceptible when mortality was 98% or higher, (2) possible resistant when mortality was between 97 and 80%, and (3) resistant when the mortality was lower than 80%. The results of the bioassays were described in Van Bortel *et al *[5]. The pyrethroid- and/or DDT-resistant *Anopheles *populations, given in Figure 1, were further analysed on the presence of *kdr *mutations. Although the *An. dirus s.s*. populations, were DDT and pyrethroid susceptible, these populations showed high levels of variation in mean knockdown time (KDT50) (ranged from 8 till 31 minutes for DDT, and from 8 till 24 minutes for pyrethroids) and were also further analysed for the presence of *kdr *mutations. After the bioassays, the mosquitoes exposed to control papers were stored in liquid nitrogen for subsequent biochemical analyses to assess the involvement of metabolic insecticide resistance mechanisms in pyrethroid- and/or DDT-resistant *Anopheles *populations. Insecticide exposed mosquitoes were dried on silica gel.

### DNA extraction

One to six legs of the dried individual mosquitoes were used for DNA extraction, applying the procedure described in Collins *et al *[13]. DNA was resuspended in 25 μl TE buffer (10 mM Tris-HCl pH 8; 1 mM EDTA). After the biochemical assays, the remaining mosquito homogenate of the control mosquitoes was spotted on filter papers. DNA of these mosquitoes was obtained by incubating a filter paper punch overnight at 4°C in 1 ml of 0.5% saponin in phosphate buffered saline (PBS). The punches were washed for 1 h in PBS at 4°C and transferred to a new tube containing 200 μl of a 20% chelex solution (Biorad, Hercules, USA). The samples were further extracted by the chelex method described in Verhaeghen *et al *[14]. A negative control was included with every set of extractions.

### Molecular identification

In the Mekong region, the three main malaria vectors *An. dirus s.l*., *An. sundaicus s.l*. and *An. minimus s.l*. are all complexes of species. Individual species within the complexes can not be distinguished on morphological characters. However, *An. dirus s.s*. and *An. epiroticus *are the only members of the *An. dirus *and *An. sundaicus *complexes observed in Cambodia and Vietnam [15,16]. *Anopheles minimus s.l*. is a complex of at least two isomorphic species, which occur in sympatry in this area [17]. Morphologically identified *An. minimus s.l*. mosquitoes were analysed by using a slightly adapted version of the PCR-RFLP developed by Van Bortel *et al *[17]. The restriction enzyme BsiZi was replaced by its isoschizomer Cfr13I. The reaction mixture contained 17 μl sterile water, 2.5 μl TangoTM buffer (provided by manufacturer), 0.5 μl of Cfr13I (10 units/μl) (Fermentas, St-Leon Rot, Germany) and 5 μl of the PCR product. The mixture was incubated for 2 h at 37°C. After incubation, the specimens were electrophoresed on a 3% mixed agarose gel (1.5% agarose and 1.5% small fragment agarose) and visualized under UV light after ethidium bromide staining.

### PCR-RFLP for the detection of *kdr *mutations in *An. dirus s.s*

The DIIS6 region of the *para*-type sodium channel gene was amplified by an adapted version of the protocol by Martinez-Torres *et al *[7]. Amplification was performed in a 50 μl reaction containing 1 μl of template DNA, 1 × Qiagen PCR buffer, 1 mM MgCl_2_, 200 μM of each dNTP, 100 nM of the primers Agd1 and Agd2 and 1 unit Taq DNA polymerase (Taq PCR core kit, Qiagen, Hilden, Germany). The cycling conditions were as follows: initial denaturation at 94°C for 3 minutes, 40 cycles of 1 minute denaturation at 94°C, 30 seconds annealing at 47°C and 30 seconds extension at 72°C followed by a final extension of 10 minutes at 72°C. Amplification products were checked on a 2% agarose gel and visualized under UV light after ethidium bromide staining. Direct PCR sequencing was performed by the VIB genetic service facility (University of Antwerp, Belgium).

Based on the DIIS6 sequence, a restriction enzyme, FspBI was found suitable to screen for the presence of a *kdr *mutation in *An. dirus s.s*. The restriction mixture contained 15.5 μl sterile distilled water, 2.5 μl buffer TangoTM, 1 μl enzyme FspBI (5'-C^TAG-3', 10 units/μl) (Fermentas, St-Leon Rot, Germany) and 6 μl of the PCR amplified template. The mixture was incubated overnight at 37°C. After incubation, the samples were electrophoresed on a 3% small fragment agarose gel and visualized.

### FRET/MCA for the detection of *kdr *mutations in *An. epiroticus, An. subpictus *and *An. minimus s.l*

Two different Fluorescence Resonance Energy Transfer/Melt Curve Analysis (FRET/MCA) assays were developed to detect *kdr *mutations in *An. epiroticus *and *An. subpictus *(FRET/MCA I) and *An. minimus s.l*. (FRET/MCA II). Those assays were adapted from the FRET/MCA developed to detect *kdr *mutations in *An. gambiae s.l*. [14].

In both assays, a primary PCR was performed with the primers Agd1 and Agd2 (FRET/MCA I) or Agd1Mi and Agd2H (FRET/MCA II) to amplify the DIIS6 region of the *para*-type sodium channel gene [7,18] (Table 1). The reaction and cycling conditions given for the amplification of the DIIS6 region of *An. dirus s.s*. were used. The DIIS6 fragment was subsequently used in an amplification reaction (20 μl), which contained 1 × iQ supermix (Biorad, Hercules, USA), 300 nM of a forward ROX labelled primer, 60 nM of a reverse primer and 1 μl of a 10-fold dilution of the primary PCR product. The PCR was performed on an iCycler with a 490/20X FAM excitation and a 620/30M ROX emission filter (Biorad, Hercules, USA) following the cycling conditions as given in Table 1. After amplification, a FAM labelled probe was added in a final concentration of 200 nM, and a melt curve was performed. Cooling to 48°C will allow the FAM labelled probe to anneal adjacent to the ROX fluorophore of the PCR product. Subsequently, the temperature is slowly increased, while the ROX fluorescence resulting from FRET, is continually monitored. When the melting temperature of the probe-amplicon hybrid is reached, the ROX fluorescence will decrease as FRET can no longer occur between the FAM label of the probe and the ROX label of the PCR product.

**Table 1:** Overview of the two FRET/MCA assays developed for detection of knockdown resistance in *An. epiroticus*, *An. subpictus*, *An. minimus s.s*. and *An. harrisoni*: sequences of oligonucleotides and cycling conditions

	**PRIMARY PCR**	**SECONDARY PCR**	**MELT CURVE ANALYSIS**
**FRET/MCA I**	**PRIMERS**	**PRIMERS**	**PROBE**
	
*An. epiroticus, An. Subpictus*	Agd1: 5'-atagattccccgaccatg-3' Agd2: 5'-agacaaggatgatgaacc-3'	sundF-ROX: 5'-tagctacggtagtgaTagg-3'* Agd2: 5'-agacaaggatgatgaacc-3'	sundF-FAM: 5'-cataccactaaatttcctaTc-3'**
	
	**CYCLING CONDITIONS**	**CYCLING CONDITIONS**	**MCA CONDITIONS**
	
	3 min denaturation at 94°C 40 cycles: 1 min denaturation at 94°C, 30 s annealing at 47°C, 30 s extension at 72°C. 10 min extension at 72°C	4 min denaturation at 95°C 30 cycles: 1 min denaturation at 95°C, 30 s annealing at 53°C,30 s extension at 72°C. 8 min extension at 72°C	1 min at 95°C 1 min at 48°C 90 repeats heating for 20 s, starting at 48°C and with 0.3°C increments.

**FRET/MCA II**	**PRIMERS**	**PRIMERS**	**PROBE**
	
*An. minimus s.l*	Agd1Mi: 5'-ctgccaagatggaattttcac-3' Agd2H: 5'-gacaaaagcaaggctaag-3'	MinF-ROX: 5'-ggctacagtagtgaTagg-3'* FunminR: 5'-gacaaaagcaaggctaagaaaag-3'	Min-FAM: 5'-cttacgactaaatttcctaTcac-3'**
	
	**CYCLING CONDITIONS**	**CYCLING CONDITIONS**	**MCA CONDITIONS**
	
	3 min denaturation at 94°C 40 cycles: 1 min denaturation at 94°C, 30 s annealing at 47°C, 30 s extension at 72°C. 10 min extension at 72°C	4 min denaturation at 95°C 30 cycles: 1 min denaturation at 95°C, 45 s annealing at 51°C, 30 s extension at 72°C. 8 min extension at 72°C	1 min at 95°C 1 min at 48°C 80 repeats heating for 15 s, starting at 48°C and with 0.3°C increments.

* ROX fluorophore on capital T, ** FAM fluorophore on capital T

The change in the ROX fluorescence appears as a positive peak on the plot of the first negative derivative of the fluorescence versus temperature function. The iCyclerTM iQ Optical system software version 3.1 was used for data analysis (Biorad, Hercules, USA) and all experiments were performed in duplicate to verify reproducibility.

Control mutant and wild type plasmids were used on each plate as positive controls. The wild type plasmids used in FRET/MCA I and FRET/MCA II originated respectively from a sequenced wild type *An. epiroticus *and *An. minimus s.s*. specimen of Vietnam. The plasmids were constructed by ligation and transformation of the primary PCR product (primers Agd1-Agd2 or Agd1Mi-Agd2H), by use of the Original TA cloning kit according to the manufacturer's instructions (Invitrogen, Carlsbad, California). For each FRET/MCA, two mutant plasmids were constructed by gene synthesis (Genscript, Piscataway, New Jersey). For the FRET/MCA I, a 153 bp fragment similar to the DIIS6 region of the *para*-type sodium channel gene of *An. epiroticus *(fragment from primer sundF-ROX to Agd2) was made. The triplet TTA, encoding the L1014 in the wild type sequence, was replaced by TCA (L1014S) or TTT (L1014F) to represent the two *kdr *mutations in *An. gambiae s.s*. The mutant plasmids used in FRET/MCA II contained a 132 bp fragment of the DIIS6 region of *An. minimus s.s*. (fragment from primer MinF-ROX to FunminR). Similarly, the triplet TTA was replaced by TCA (L1014S) or TTT (L1014F). All mutant plasmids were cloned in a pUC57 vector and transformed in TOP10 cells (Invitrogen, Carlsbad, California). Five millilitres of each clone was purified on a column (QIAprep Spin Miniprep kit, Qiagen, Hilden, Germany) and DNA was resuspended in 50 μl water. One microlitre of a 100-fold dilution of the plasmids was used in the secondary PCR of the FRET/MCA assay.

### Biochemical assays

In order to obtain a complete resistance profile for the main malaria vectors, biochemical assays were performed on resistant *An. minimus s.l*., *An. epiroticus *and *An. subpictus *populations of Vietnam. The main vector *An. dirus s.l*. could not be tested in the biochemical assays because the collection sites in Central Vietnam could not be accessed with a liquid nitrogen container. Only mosquitoes exposed to control papers during the WHO bioassays were used for these assays.

To ensure that the presence of blood could not interfere with the biochemical assays, the abdomen of the individual mosquitoes was removed. The remaining head-thorax portion was homogenized in 200 μl distilled water and centrifuged at 13,000 g for 2 min. The monooxygenase, esterase (para-nitrophenyl acetate as substrate), GST (1-chloro-2,4-dinitrobenzene as substrate) and protein assay were carried out, in duplicate, on the supernatant as described by Penilla *et al *[19].

For this study, no fully susceptible reference colony strains for different test species were available. Therefore, an *An. minimus s.s*. field population of VHBA (Hoa Binh Province, northern Vietnam; figure 1), fully susceptible in the WHO bioassays, was taken as reference. The two-sample Kolmogorov-Smirnov Z test (SPSS 14) was used to compare the results of the biochemical assays with the reference *An. minimus s.s*. population of VHBA.

## Results

### Molecular identification

*Anopheles minimus s.l*. is a complex of at least two isomorphic species, which occur in sympatry in Vietnam [17]. All *An. minimus s.l *specimens used to study target-site insensitivity and metabolic resistance, were molecular identified (Table 2). In Cambodia, most of the specimens identified as *An. minimus s.l*. (60 of 62; 96.8%) proved to be *An. minimus s.s*. In Vietnam, 2,425 of the 2,480 (97.8%) morphologically identified *An. minimus s.l*. specimens belong to the species complex. Among the mosquitoes that survived the bioassay test, both species, *An. minimus s.s*. and *Anopheles harrisoni*, were found, relative to the proportion found in the entire population of that site.

**Table 2:** Molecular identification of morphologically identified *An. minimus s.l*. specimens

Country	Morphological identification	Molecular identification				
		
		*An. minimus s.s*.	*An. harrisoni*	*An. jeyporiensis*	*An. aconitus*	others
Cambodia	*An. minimus s.l*.	60				2
Vietnam	*An. minimus s.l*.	1873	552	27	20	8

### Detection of *kdr *mutations

**Figure 2:**
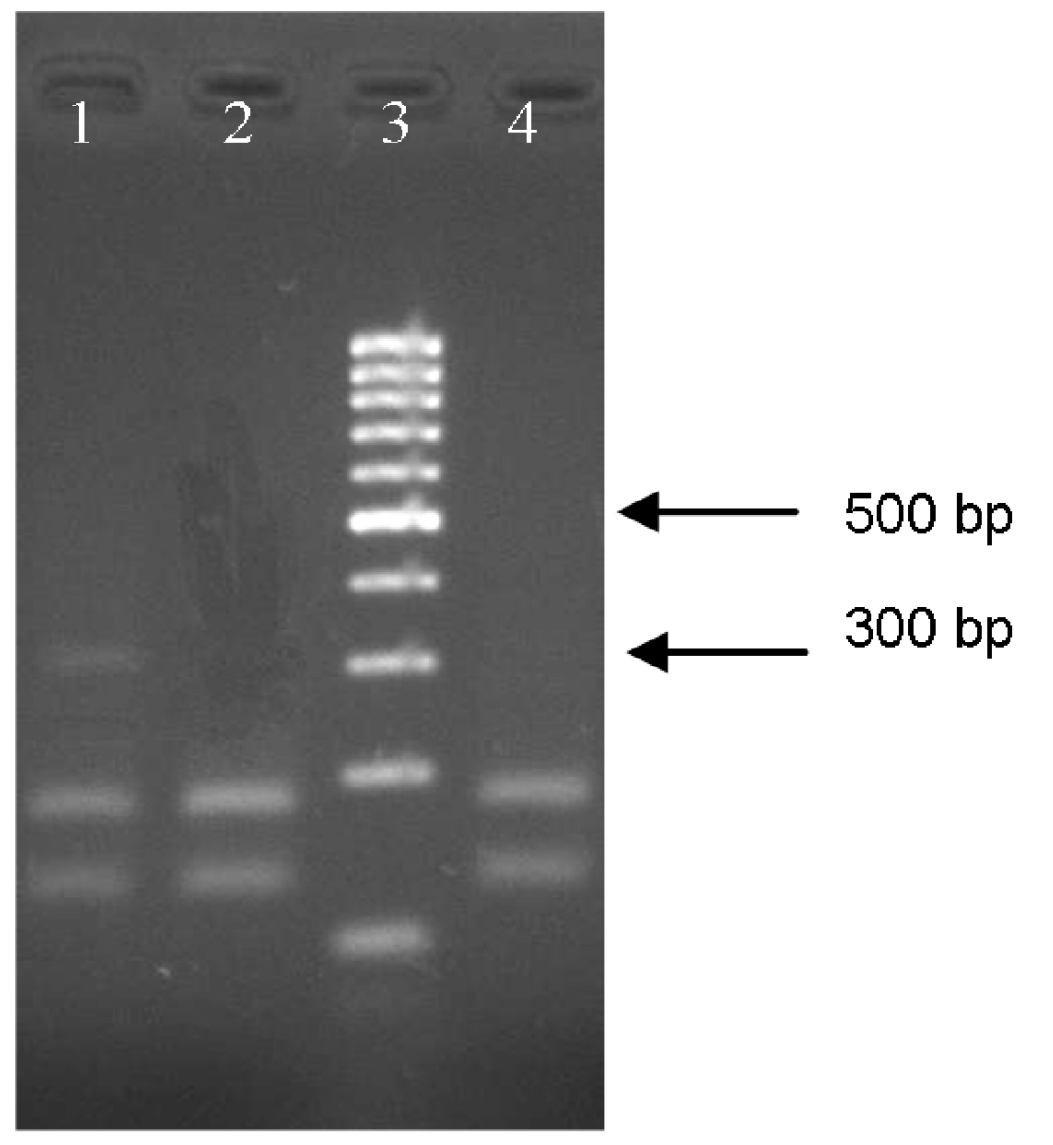
**PCR-RFLP for the detection of *kdr *mutations in *An. dirus s.s***. Restriction patterns obtained by PCR-RFLP after separation on a 3% small fragment agarose gel. Lane 1: *An. dirus s.s*. with incomplete digestion; Lane 2 and 4*: An. dirus s.s*. with complete restriction; Lane 3: 100 bp ladder.

By use of the primers Agd1 and Agd2, a 301 bp genomic DNA fragment of the DIIS6 region was obtained for *An. dirus s.s*. [GenBank:EU155383]. This genomic DNA fragment showed 82% sequence identity to the corresponding genomic DNA fragment of the *para*-type sodium channel gene of *An. gambiae s.s*. In *An. dirus s.s*., the leucine at codon 1014 is encoded by the triplet CTA. The restriction enzyme FspBI recognizes the palindrome CTAG and cuts the 301 bp sequence of the *para*-type sodium channel gene of wild type *An. dirus s.s*. specimens into two fragments with a length of 127 bp and 174 bp (Figure 2). In total, 927 *An. dirus s.s*. specimens (Table 3) originated from nine Cambodian and 11 Vietnamese collection sites were tested with this PCR-RFLP. Of these 927 specimens, 854 showed a complete digestion corresponding to the wild type triplet (CTA), whereas 73 specimens showed an incomplete digestion with the restriction enzyme FspBI. The partial restriction occurred among control, alive and death insecticide exposed mosquitoes. The DIIS6 region of the *para*-type sodium channel gene was sequenced for all specimens with a partial digestion, but no *kdr *mutation was observed among these 73 specimens.

**Table 3:** Number of specimens (n) analysed for the presence of *kdr *with the developed PCR-RFLP or the FRET/MCA assays

Species	Technique	N	Insecticide exposed		Control
				
			Alive	Dead	
*An. dirus s.s*.	PCR-RFLP	927	64	668	195

*An. epiroticus*	FRET/MCA I	995	182	151	662
*An. subpictus*	FRET/MCA I	220	85	77	58

*An. minimus s.s*.	FRET/MCA II	1934	612	1005	317
*An. harrisoni*	FRET/MCA II	553	118	335	100

**Figure 3:**
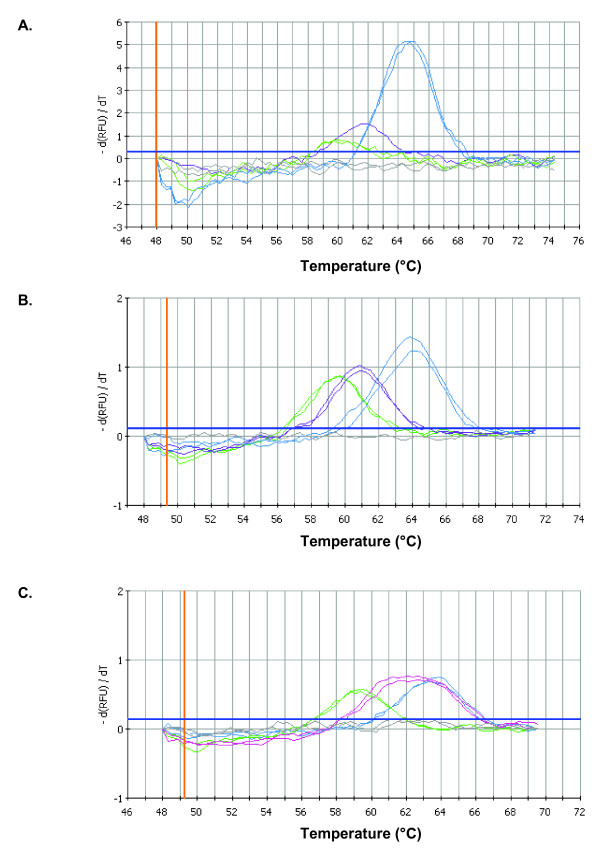
**FRET/MCA for detection of *kdr *mutations in *An. epiroticus *and *An. minimus s.l***. The FRET/MCA of the DIIS6 region of the *para*-type sodium channel gene plotted as the first negative derivative of the relative fluorescence unit (-d(RFU)/dT) versus temperature function. In all panels, the thicker blue horizontal line denotes the threshold for background fluorescence, and the curve entirely below denotes the results for the non-template control (gray). (A) FRET/MCA I *An. epiroticus *plasmids, from low to high Tm: the L1014S (green), the L1014F (purple) and wild type L1014 plasmid (blue). Melting peaks were identified at 60.3°C, 61.8°C and 64.5°C, respectively. (B) FRET/MCA II *An. minimus s.s*. plasmids, from low to high Tm: the L1014S plasmid (green), Tm of 59.7°C; the L1014F plasmid (purple), Tm of 60.9°C; and the wild type L1014 plasmid (blue), Tm of 63.9°C. (C) FRET/MCA II from low to high Tm: the L1014S plasmid (green), Tm of 59.7°C; the *An. harrisoni *mosquito of VLSB with a different melting peak with Tm of 62.1°C (pink); and wild type *An. harrisoni *specimens (blue), Tm of 63.9°C.

The same primers, Agd1 and Agd2, amplified a 303 bp genomic DNA fragment of the DIIS6 region for *An. epiroticus *[GenBank: EU155384] and *An. subpictus *[GenBank: EU155385]. The fragments showed respectively, 87% and 86% sequence identity to the corresponding genomic DNA fragment of *An. gambiae s.s*. This 303 bp fragment was further used as template in the FRET/MCA I. For *An. epiroticus*, the FRET/MCA results in melt curves with a melting temperature (Tm) of approximately 60.3°C, 61.8°C and 64.5°C for the constructed L1014S and L1014F *kdr *plasmids and the wild type *An. epiroticus *plasmid, respectively (Figure 3A). Wild type *An. subpictus *produced the same melt curves as wild type *An. epiroticus *because the sequence of the *para*-type sodium channel gene at the hybridization place of the probe is identical. In total, 995 *An. epiroticus *and 220 *An. subpictus *mosquitoes were analysed (Table 3). All samples were characterized by a melt peak with a Tm of 64.5°C, which corresponded to the susceptible genotype. No *kdr *mutation was observed for *An. epiroticus *or *An. subpictus*.

**Figure 4:**
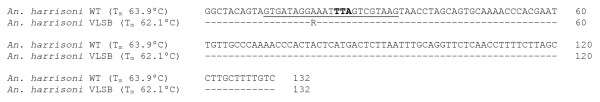
**Sequences of the DIIS6 region of the *para*-type sodium channel gene obtained for *An. harrisoni *mosquitoes with respectively a Tm of 63.9°C (wild type, WT) and 62.1°C**. Sequences of the 132 bp fragment of theDIIS6 region of the *para*-type sodium channel gene obtained after amplification with primers MinF-ROX and FunminR of *An. harrisoni *mosquitoes with respectively a Tm of 63.9°C (wild type, WT) [GenBank: EU155387] and a Tm of 62.1°C (VSLB) [GenBank: EU258605] in the FRET/MCA. The triplet TTA at codon 1014 is given in bold, while the hybridization place of the probe is underlined. A line (-) indicates a nucleotide identity compared to the wild type *An. harrisoni *sequence. R = heterozygote A/G

The primers Agd1Mi and Agd2H amplified for *An. minimus s.s*. [GenBank: EU155386] and *An. harrisoni *[GenBank: EU155387] a 263 bp genomic DNA fragment with 85% sequence identity to the corresponding genomic DNA fragment of *An. gambiae s.s*. In the FRET/MCA II, melt curves with melting temperatures of 59.7°C, 60.9°C and 63.9°C were obtained for respectively the L1014S, the L1014F *kdr *plasmids and the wild type *An. minimus s.s*. plasmid (Figure 3B). Wild type *An. harrisoni*, produced the same melt curves as the wild type *An. minimus s.s*., because at the hybridization place of the probe the sequences are identical. By use of the FRET/MCA II, 1934 *An. minimus s.s*. and 553 *An. harrisoni *mosquitoes were analysed on the presence of *kdr *mutations (Table 3). One *An. harrisoni *mosquito used as control in the WHO bioassay and originated from VLSB (Long Son Province, northern Vietnam) produced in the FRET/MCA a broad melt peak with a Tm of 62.1°C, which was different from the expected Tm of the wild type or mutant alleles (Figure 3C). Sequencing of the 132 bp fragment of domain II of the *para*-type sodium channel gene revealed that this specimen possessed at one allele a single nucleotide polymorphism, four nucleotides before codon 1014 (Figure 4). Additional tests done on the closely related *Anopheles jeyporiensis *(n = 294) [GenBank: EU155389] and *Anopheles aconitus *(n = 252) [GenBank: EU155388] demonstrated that the FRET/MCA II can be used to detect *kdr *mutations in these species.

### Biochemical assays

Non-specific esterases, monooxygenase and GST assays were performed on different *Anopheles *populations of Vietnam (Table 4). A significant increase in esterase activity was measured in the *An. minimus s.s*. populations from northern Vietnam (VLSA, VNAB, VQNA, VQNB). All these populations were possibly pyrethroid-resistant in the WHO bioassays (Table 5). The pyrethroid susceptible *An. minimus s.s*. populations did not show a significantly increased esterase activity. The enzyme levels of the Vietnamese *An. harrisoni *populations were not significantly increased, although the VLSB *An. harrisoni *population was pyrethroid-resistant in the WHO bioassay (Table 5).

**Table 4:** Mean and standard error (SE) obtained for the esterase, monooxygenase and GST assay on different Vietnamese populations of *An. minimus s.l*., *An. epiroticus *and *An. subpictus*.

Species	Study site and year	n	Esterase (PNPA) (μmol/min/mg protein)			Monooxygenase (nmol equivalent unit cyt P450/mg protein)			GST (CDNB) (mmol/min/mg protein)		
			
			Mean	SE	p-value^1^	Mean	SE	p-value^1^	Mean	SE	p-value^1^
*An. minimus s.s*.	VHBA 2003	36	0.0615	0.0053		0.5877	0.0626		0.1324	0.0182	
	VHBA 2004	25	0.0484	0.0054	0.180	0.5337	0.0524	0.232	0.1409	0.0192	0.611
	VHBB 2003	40	0.0693	0.0101	↓ 0.001	0.6036	0.0306	0.177	0.1296	0.0165	0.858
	VHBB 2004	40	0.0585	0.0063	0.066	0.6317	0.0412	0.321	0.1208	0.0128	0.383
	VTHA 2004	26	0.0565	0.0088	↓ 0.011	0.1771	0.0210	↓ 0.000	0.1534	0.0169	0.181
	VLSA 2004	44	0.0969	0.0070	↑ **0.000**	0.1551	0.0144	↓ 0.000	0.0970	0.0117	0.075
	VQNA 2004	29	0.1071	0.0112	↑ **0.001**	0.2749	0.0206	↓ 0.000	0.0744	0.0121	↓ 0.047
	VQNB 2004	30	0.1081	0.0056	↑ **0.000**	0.2749	0.0291	↓ 0.000	0.0829	0.0111	0.053
	VNAB 2004	12	0.1486	0.0156	↑ **0.000**	1.1702	0.1799	↑ **0.002**	0.0590	0.0147	0.057
											
*An. harrisoni*	VSLA 2003	27	0.0602	0.0045	0.065	0.5916	0.0433	0.726	0.0492	0.0090	↓ 0.002
	VSLB 2003	29	0.0607	0.0033	0.084	0.6277	0.0408	0.072	0.0637	0.0116	↓ 0.009
	VLSB 2004	26	0.0418	0.0065	↓ 0.000	0.2865	0.0383	↓ 0.000	0.1567	0.0293	0.174
											
*An. epiroticus*	VKGA 2003	30	0.0773	0.0080	↑ **0.032**	0.0916	0.0087	↓ 0.000	0.1172	0.0124	0.282
	VHCA 04/2003	30	0.0953	0.0137	↑ **0.025**	0.2257	0.0126	↓ 0.000	0.1691	0.0272	0.604
	VHCB 2003	30	0.0898	0.0126	0.177	0.1861	0.0084	↓ 0.000	0.1618	0.0175	0.059
	VBTA 2003	27	0.1304	0.0160	↑ **0.000**	0.1532	0.0134	↓ 0.000	0.2163	0.0307	0.111
	VBLB 2003	27	0.1082	0.0069	↑ **0.000**	0.2131	0.0167	↓ 0.000	0.1391	0.0171	0.065
	VHCA 10/2003	9	0.1956	0.0300	↑ **0.000**	0.1295	0.0074	↓ 0.000	0.1036	0.0214	0.759
	VBLA 2003	44	0.0955	0.0084	↑ **0.004**	0.2015	0.0262	↓ 0.000	0.1249	0.0162	0.528
	VBLA 2004	30	0.1008	0.0125	↑ **0.006**	0.1823	0.0142	↓ 0.000	0.1053	0.0165	0.641
	VBLB 2004	22	0.1199	0.0104	↑ **0.000**	0.1670	0.0093	↓ 0.000	0.1321	0.0260	0.361
	VBTB 2003	29	0.1247	0.0139	↑ **0.000**	0.1793	0.0148	↓ 0.000	0.1409	0.0219	0.146
	VCMA 2003	30	0.0754	0.0087	**↑ 0.020**	0.0952	0.0074	↓ 0.000	0.0906	0.0113	↓ 0.036
	VCMB 2003	30	0.1344	0.0132	↑ **0.000**	0.1454	0.0140	↓ 0.000	0.0970	0.0134	0.075
	VCMC 2003	26	0.2494	0.0336	↑ **0.000**	0.2981	0.0181	↓ 0.000	0.1492	0.0138	0.117
	VCMD 2003	13	0.1703	0.0329	↑ **0.001**	0.2793	0.0221	↓ 0.001	0.1700	0.0265	0.220
	VKGS 2003	30	0.0867	0.0088	↑ **0.036**	0.0936	0.0155	↓ 0.000	0.1308	0.0192	0.967
	VLAA 2003	20	0.0919	0.0114	↑ **0.029**	0.1419	0.0062	↓ 0.000	0.1534	0.0147	0.063
	VLAB 2003	30	0.0714	0.0084	**↑ 0.008**	0.0839	0.0117	↓ 0.000	0.1105	0.0087	↓ 0.014
	VSTA 2003	30	0.0940	0.0076	↑ **0.000**	0.2040	0.0094	↓ 0.000	0.1292	0.0103	0.075
	VTGA 2003	37	0.0822	0.0082	↑ **0.015**	0.1135	0.0131	↓ 0.000	0.0732	0.0100	↓ 0.029
											
*An. subpictus*	VLAA 2003	30	0.0715	0.0080	0.130	0.2782	0.0353	↓ 0.000	0.2938	0.0305	↑ **0.001**
	VKGB 2003	17	0.1300	0.0213	↑ **0.003**	0.1324	0.0123	↓ 0.000	0.3375	0.0248	↑ **0.000**

↑ Mean rank of population is increased compared to the *An. minimus s.s*. population of VHBA.

↓ Mean rank of population is decreased compared to the *An. minimus s.s*. population of VHBA.

^1 ^P values (Two-sample Kolmogorov-Smirnov Z) indicating a significant increased level of esterase activity, GST activity or an elevated level of monooxygenase compared to the *An. minimus s.s*. population of VHBA are given in bold

The esterase activity was measured with para-nitrophenyl acetate (PNPA), whereas the GST activity was measured with 1-chloro-2,4-dinitrobenzene (CDNB) as substrate.

**Table 5:** Overview of the possible metabolic resistance mechanisms in relation to the mortality categories in the WHO bioassay as published in [5].

Species	Study site and year	BIOASSAYS (mortality categories)^1^				BIOCHEMICAL ASSAYS^2^		
		
		DDT 4%	permethrin 0.75%	Alpha-cypermethrin 0.082%	Lambda-cyhalothrin 0.05%	esterases	Mono-oxygenases	GST
*An. minimus s.s*.	VHBA, 2003	S	S	S	S	-	-	-
	VHBA, 2004	S	S	S	S	NO	NO	NO
	VHBB, 2003	S	S	S	S	NO	NO	NO
	VHBB, 2004	S	S	S	S	NO	NO	NO
	VTHA, 2004	S	S	S	PR	NO	NO	NO
	VLSA, 2004	S	PR	PR	PR	YES	NO	NO
	VQNA, 2004	S	PR	PR	R	YES	NO	NO
	VQNB, 2004	S	PR	R	R	YES	NO	NO
	VNAB, 2004	nd	Nd	PR	nd	YES	YES	NO
*An. harrisoni*	VSLA, 2003	PR	S	nd	S	NO	NO	NO
	VSLB, 2003	S	S	nd	nd	NO	NO	NO
	VLSB, 2004	nd	R	PR	PR	NO	NO	NO
*An. epiroticus*	VKGA, 2003	S	PR	R	R	YES	NO	NO
	VHCA, 04/2003	S	PR	R	R	YES	NO	NO
	VHCB, 2003	PR	PR	R	R	NO	NO	NO
	VBTA, 2003	PR	PR	PR	PR	YES	NO	NO
	VBLB, 2003	nd	PR	nd	R	YES	NO	NO
	VHCA, 10/2003	PR	R	R	R	YES	NO	NO
	VBLA, 2003	S	R	PR	PR	YES	NO	NO
	VBLA, 2004	S	R	S	R	YES	NO	NO
	VBLB, 2004	S	R	R	R	YES	NO	NO
	VBTB, 2003	S	R	PR	R	YES	NO	NO
	VCMA, 2003	S	R	R	PR	YES	NO	NO
	VCMB, 2003	S	R	R	R	YES	NO	NO
	VCMC, 2003	S	R	R	R	YES	NO	NO
	VCMD, 2003	S	R	R	R	YES	NO	NO
	VKGS, 2003	S	R	R	R	YES	NO	NO
	VLAA, 2003	S	R	R	R	YES	NO	NO
	VLAB, 2003	S	R	PR	R	YES	NO	NO
	VSTA, 2003	S	R	PR	R	YES	NO	NO
	VTGA, 2003	PR	R	nd	Nd	YES	NO	NO
*An. subpictus*	VLAA, 2003	PR	PR	PR	PR	NO	NO	YES
	VKGB, 2003	PR	R	PR	R	YES	NO	YES

^1^Mortality categories

S Susceptible (mortality > 98%)

PR Possibility of resistance (mortality between 80–97%)

R Resistant (mortality < 80%)

Nd Not done

^2^YES: mean rank of population is significantly higher than mean rank of VHBA *An. minimus s.s*. population.

NO: mean rank of population is not significant higher than mean rank of VHBA *An. minimus s.s*. population

All *An. epiroticus *populations of the Mekong Delta, except the population of VHCB, had an increased esterase activity (1.5 to 4 fold higher) compared to the *An. minimus s.s*. population of VHBA (Table 4). In the bioassays, these populations were characterized by pyrethroid resistance (Table 5). In *An. subpictus*, the esterase activity was significantly higher (2 times higher) in the VKGB population. The esterase activity was not significantly increased in the possible permethrin-resistant (97% mortality) VLAA *An. subpictus *population (Table 5).

Almost no differences in GST activity were observed between the different *Anopheles *populations. Only, the possible DDT-resistant *An. subpictus *populations from VKGB and VLAA were characterized by a significant higher GST activity (2.5 fold higher) (Tables 4 and 5).

In general, the estimates of the monooxygenase levels in *An. minimus s.l*., *An. epiroticus *and *An. subpictus *populations were significantly lower than those obtained for *An. minimus s.s*. of VHBA. A significant increased monooxygenase level (2 fold) was only detected in the *An. minimus s.s*. population from VNAB (Table 4). In the WHO bioassay, this population was only tested with alpha-cypermethrin and had a 24 h mortality of 90% (Table 5).

## Discussion

In the 1960s, malaria control in Vietnam was primarily based on the use of DDT for residual house spraying. Twenty years later, DDT resistance emerged in the malaria vectors of the coastal areas, *An. epiroticus *and *An. subpictus *[20]. Nowadays, the use of DDT in the malaria control programme of Vietnam is completely stopped for about 20 years [20] and bioassays conducted from 2003 until 2005 revealed that most *Anopheles *species, except *An. subpictus *and *An. epiroticus *near Ho Chi Minh City, were susceptible to DDT [5]. This suggests that the insecticide resistance may decrease or even disappear if the pressure of the concerned insecticide does not exist for a long period. Pyrethroid insecticides replaced DDT in the malaria vector control [21]. In 2005, *An. epiroticus *and *An. subpictus *of the Mekong Delta were highly pyrethroid-resistant. *An. minimus s.l*. was pyrethroid-resistant in northern Vietnam, whereas *An. dirus s.s*. was possibly resistant to type II pyrethroids in central Vietnam [5].

Insects may survive the toxic effect of insecticides by different physiological mechanisms including target-site insensitivity and elevated detoxifying enzyme production. Knockdown resistance, caused by a mutation at codon 1014 of the *para*-type sodium channel gene, is described in several *Anopheles *species including *An. gambiae s.s*., *Anopheles arabiensis*, *Anopheles culicifacies*, *Anopheles stephensi*, *Anopheles sinensis*, *Anopheles sacharovi *and *An. subpictus *[7; 22-27]. Here, a PCR-RFLP was developed to study knockdown resistance in *An. dirus s.s*. In this species the triplet encoding the wild type leucine at codon 1014 is CTA. This triplet allows the screening of *An. dirus s.s*. populations for *kdr *alleles by using the restriction enzyme FspBI, which will cut the 301 bp of the *para*-type sodium channel gene of wild type *An. dirus s.s*. specimens into two fragments. In addition to the PCR-RFLP, two FRET/MCA assays were developed to study knockdown resistance in *An. epiroticus*, *An. subpictus *and *An. minimus s.l*. As was demonstrated by mutant plasmids, SNPs occuring at the probe-amplicon hybrid of the DIIS6 region of the *para*-type sodium channel gene can be detected with this technique. A large number of *Anopheles *populations of the Mekong region characterized by a high survival rate or a prolonged mean knockdown time in the WHO bioassay were tested by use of the FRET/MCA or PCR-RFLP on the presence of *kdr *mutations, but no *kdr *mutation was observed in any of the main vectors. As with the FRET/MCA and the PCR-RFLP only mutations at respectively the probe-amplicon hybrid or codon 1014 can be detected, we cannot exclude the presence of other mutations in the *para*-type sodium channel gene. However, the almost absence of DDT resistance in *An. minimus s.l*., *An. epiroticus *and *An. dirus s.s*. indicated that knockdown resistance is unlikely and that other resistance mechanisms might be involved.

As *kdr *mutations have arisen rapidly and repeatedly in many other pyrethroid and/or DDT resistant *Anopheles *species of different geographical areas, the absence of this mutation in the main *Anopheles *vectors of the Mekong region might reflect a different insecticidal pressure and/or genetic constraint. The triplet present at codon 1014 might already explain why no *kdr *mutation was found for *An. dirus s.s*. For this species, the probability of finding a known *kdr *associated amino acid at codon 1014 (phenylalanine, serine, histidine or cysteine) is lowered, as at least two nucleotide substitutions are required in the codon CTA to obtain a change to a phenylalanine, serine, histidine or cysteine. However, it should also be pointed out that there are examples of double nucleotide point mutations conferring target site insensitivity, for example the serine (TCA) to phenylalanine (TTT) substitution at codon 431 of an acetylcholinesterase gene found in *Myzus persicae *[28] and the leucine (TTG) to cysteine (TGT) mutation at codon 1014 of the *para*-type sodium channel gene in permethrin-resistant *An. sinensis *[27]. In addition, it should be noticed that although the *kdr *mutation was not observed in the Vietnamese *An. subpictus*, a L1014F *kdr *mutation was found in a pyrethroid-resistant *An. subpictus *population of Sri Lanka [26]. However, as different sibling species of *An. subpictus *may exist in Sri Lanka [29], the presence of knockdown resistance might be limited to one species of the complex. Otherwise, the two *An. subpictus *populations from Vietnam and Sri Lanka are geographically isolated which may prevent gene flow [26].

Because no *kdr *mutation was observed in the main vectors of the Mekong region, metabolic resistance mechanisms were studied by use of biochemical assays. Biochemical assays point towards metabolic resistance mechanisms, by indicating whether there is a general P450 monooxygenase, glutathione-S-transferase or esterase response. High GST activities were found in *An. subpictus*. Glutathione-S-transferases catalyse the dehydrochlorination of DDT to DDE and have been reported to play a significant role as DDT resistance mechanism in many insects including *An. gambiae *and *Anopheles crascens *of Thailand [30,31]. In addition, GSTs can mediate insecticide resistance by conjugation of glutathione to the insecticide or its primary toxic metabolic product. GSTs were found to play a minor role as pyrethroid resistance mechanism in *An. funestus *[11]. Because *An. subpictus *was possible DDT-resistant, it is likely that the high GST activity detected in this study confers DDT resistance. High esterase activities were measured in the pyrethroid-resistant *An. epiroticus *of the Mekong Delta, in *An. minimus s.s*. from northern Vietnam and in *An. subpictus *of southern Vietnam. A combination of a high esterase activity and an elevated P450 monooxygenase level was detected in an *An. minimus s.s*. population of northern Vietnam (VNAB). However, for the *An. minimus s.s*. population of VNAB no complete resistance profile was obtained. In general, P450 monooxygenases can mediate resistance to all classes of insecticides whereas esterases can mediate resistance to organophosphates, carbamates and pyrethroids which are rich with ester-bonds. In insecticide-resistant mosquitoes, elevated levels of P450 monooxygenases and esterases were frequently linked to pyrethroid resistance. For example, Vulule *et al*. [10] demonstrated elevated monooxygenase and esterase levels in permethrin-resistant *An. gambiae s.s*. from Kenya. Brogdon *et al*. [9,32] reported monooxygenase and esterase based resistance mechanisms alone or in combination, in permethrin-resistant *Anopheles albimanus *from Guatemala. In an *An. minimus s.s*. colony of Thailand, deltamethrin-resistant was primarily associated with increased detoxification by P450 monooxygenases [33].

Many biological processes may affect gene expression making the interpretation of biochemical assays not straightforward. It is always possible that the measured insecticide detoxification levels represent other processes than insecticide resistance. Therefore biochemical assays should only be used to formulate hypothesis on the possible metabolic resistance mechanisms involved. Synergist studies should be further performed to confirm the role of the detected metabolic enzyme levels in the detoxification of pyrethroids and/or DDT. In addition, as our biochemical assays were performed on field populations of *Anopheles *species; differences in species, age and blood feeding status might induce an extra variability in the metabolic enzyme data [34,35]. To account for differences in enzyme levels due to age, WHO recommends using non-blood fed, adult mosquitoes of the same age, which can only be obtained by collecting larvae and rearing to adults. However, collecting an appropriate number of larvae of the major *Anopheles *species in the Mekong region is problematic due to the scattered nature of their breeding sites. In the current study, biochemical assays were performed on field collected adults used as control in the WHO tube bioassay. To ensure that the presence of blood could not interfere in the biochemical assays, the abdomen of the mosquitoes was removed. Despite the limitations of these biochemical assays, the presence of a systematic increase of a certain enzyme system in a large number of DDT and/or pyrethroid-resistant *Anopheles *populations of a certain species (e.g. systematic increase of esterases in pyrethroid- resistant *An. epiroticus*) might indicate the involvement of this metabolic enzyme system as insecticide resistance mechanism.

Another drawback of the biochemical assays was the absence of fully susceptible reference strains for each species tested. The *An. minimus s.s*. population collected in 2003 in the Hoa Binh province was used as reference strain based on the bioassay results (100% mortality against DDT, permethrin, alpha-cypermethrin and lambda-cyhalothrin), but compared to the other field populations, this *An. minimus s.s*. population had relative high levels of P450 monooxygenases. Significant lower levels of P450 monooxygenases were measured in the *An. epiroticus *and *An. subpictus *populations. In fully susceptible strains of *An. gambiae s.s*. and *An. albimanus*, the monooxygenase levels were also lower than the levels measured in the VHBA *An. minimus s.s*. population [19,36]. Additionally, WHO bioassays carried out in 2004 on 1–2 days old mosquitoes of the VHBA *An. minimus s.s*. population showed reduced mortality against the type II pyrethroids (alpha-cypermethrin 96% and lambda-cyhalothrin 94% mortality) and the non-ester pyrethroid etofenprox (95% mortality). Taking this into account, it is likely that the pyrethroid resistance in *An. minimus s.l*. could be conferred to an increased detoxification by both P450 monooxygenases and esterases, whereas in *An. epiroticus *and *An. subpictus *the pyrethroid resistance could be conferred to an esterase mediated detoxification. However, additional WHO bioassays performed on 1–2 days old *An. epiroticus *mosquitoes of VBLA and VBLB (Mekong Delta) revealed a low mortality against the non-ester pyrethroid etofenprox [5]. This means that it is likely that beside an esterase mediated detoxification also other pyrethroid resistance mechanisms are involved in *An. epiroticus *of the Mekong Delta.

Knowledge on the different resistance mechanism is necessary to guide the insecticide use in the vector control programmes as each insecticide resistance mechanism may have a different impact on the effectiveness of the pyrethroid based control programmes. Studies in West Africa have shown that ITNs remain effective against *kdr*-resistant *An. gambiae s.s*. [37-39]. Chandre *et al *[40] demonstrated that large proportions of *kdr *homozygous resistant (L1014F/L1014F) females were killed by prolonged contact with pyrethroids due to diminished sensitivity to the excito-repellent effect of the insecticide. However, recent studies have shown a reduced efficacy of ITNs when the L1014F *kdr *allelic frequency is high [41,42]. In South Africa, metabolic pyrethroid resistance in *An. funestus *required a switch back from pyrethroid insecticides to DDT for house spraying to restore the malaria vector control [43]. Elevated levels of metabolic enzymes in *An. gambiae s.s*. of Cameroon did not influence the personal protection afforded by ITNs, but did reduce the vector mortality which could in turn limit the mass effect [36]. As in the Mekong region, metabolic resistance mechanisms are involved in the main malaria vectors, the operational impact should be further studied as it may have implications for the sustained efficacy of the ITN based control programmes. As different metabolic enzyme systems might be responsible for the pyrethroid and DDT resistance in the main vectors, each species may have a different response to alternative insecticides. Furthermore, assessing the susceptibility status of the main malaria vectors to organophosphates and carbamates will provide information on the usefulness of these insecticides in the vector control programmes.

## Conclusion

A three-year survey on insecticide resistance in the main malaria vectors of the Mekong region showed that the main vector *An. epiroticus *was pyrethroid-resistant in the Mekong delta. *Anopheles minimus sensu lato *was pyrethroid-resistant in northern Vietnam, whereas *An. dirus sensu stricto *was possible resistance to type II pyrethroids in central Vietnam. The secondary vector *An. subpictus *was both DDT and pyrethroid-resistant in the Mekong Delta. As the operational implications of insecticide resistance will largely depend on the resistance mechanisms involved, the role of both knockdown and metabolic resistance was assessed in the main vectors of the Mekong region. Two FRET/MCA assays and one PCR-RFLP were developed to detect *kdr *mutations, but no *kdr *mutation was observed in any of these vectors albeit many samples of different populations from a wide geographical area were screened. Biochemical assays suggested the involvement of metabolic resistance mechanisms. As different metabolic enzyme systems might be responsible for the pyrethroid and DDT resistance in the main vectors, each species may have a different response to alternative insecticides, which might complicate the malaria vector control in the Mekong region.

## Competing interests

The authors declare that they have no competing interests.

## Authors' contributions

KV developed the *kdr *detection methods, carried out the molecular detection of the *kdr *mutations and the biochemical assays and drafted the manuscript. WVB and MC designed the study and supervised the work critically at all stages. HT and TS facilitated and carried out the field work.
